# Ethnomedicinal, Phytochemical and Pharmacological Profile of *Anthriscus sylvestris* as an Alternative Source for Anticancer Lignans

**DOI:** 10.3390/molecules200815003

**Published:** 2015-08-17

**Authors:** Octavian Tudorel Olaru, George Mihai Niţulescu, Alina Orțan, Cristina Elena Dinu-Pîrvu

**Affiliations:** 1 Carol Davila University of Medicine and Pharmacy, Traian Vuia 6, Bucharest 020956, Romania; E-Mails: octav_olaru2002@yahoo.com (O.T.O); ecristinaparvu@yahoo.com (C.E.D.-P); 2 Faculty of Biotechnology, University of Agricultural Sciences and Veterinary Medicine, Bucharest 020956, Romania; E-Mail: alina_ortan@hotmail.com

**Keywords:** wild chervil, deoxypodophyllotoxin, etoposide, teniposide, topoisomerase inhibitor, flavonoids, antimicrobial, anti-inflammatory, antioxidant

## Abstract

*Anthriscus sylvestris* (L.) Hoffm. is a wild herbaceous plant common in most temperate regions. It has been used traditionally to treat headaches, as a tonic, as antitussive, antipyretic, analgesic and diuretic. The plant contains deoxypodophyllotoxin, which is proven to have antitumor and anti-proliferative effects, anti-platelet aggregation, antiviral, anti-inflammatory and insecticidal activity. Deoxypodophyllotoxin is considered to be the plant’s most important constituent, because of its pharmacological properties and because it can be converted into epipodophyllotoxin, the main raw material for the semisynthesis of the cytostatic agents etoposide and teniposide. This work summarizes for the first time the results related to the botanical description, distribution and habitat, phytochemical and pharmacological properties and emphasizes the aspects for future biotechnological research to establish its utility in the therapeutic arsenal.

## 1. Introduction

*Anthriscus sylvestris* (L.) Hoffm., known as wild chervil or cow parsley, belongs to the Apiaceae family and is a wild plant common in most temperate regions. Related members of the Apiaceae include anise, carrot, celery, chervil, coriander, cumin, fennel, hemlock and parsley [1]. It is most commonly found in hedgerows and road verges, but also on woodland edges, neglected pastures and hay meadows [2]. It have been used traditionally as antitussive, antipyretic, analgesic, diuretic, and a cough remedy [3]. It has been demonstrated to possess deoxypodophyllotoxin, which is described to have antitumor and anti-proliferative effects [4], anti-platelet aggregation activity [5], antiviral [6], anti-inflammatory [7] and insecticidal activity [3] in several *in vitro* tests. Deoxypodophyllotoxin is considered to be the plant’s most important constituent, due to its pharmacological properties and because it can be used for the semisynthesis of podophyllotoxin and of the related cytostatic agents etoposide and teniposide [8].The importance of *A. sylvestris* as a deoxypodophyllotoxin resource is justified because of the lignan’s scarcity in natural sources and the very difficult and expensive synthesis procedures[8].The present review covers the available literature and offers systematic information for further studies on *A. sylvestris* and also on its main lignan constituent.

## 2. Botany

### 2.1. Taxonomical Classification

*A. sylvestris* belongs to the Apiaceae (syn. Umbelliferae) family, tribe *Scandiceae* Drude, section *Cacosciadium* Rchb [9]. The taxonomic classification, initially made intuitively by Drude rather than on synapomorphies [10], was confirmed after more than a hundred years by Downie *et al.* by cladistic analysis of molecular data [11]. Numerous literature sources mention only *Chaerefolium silvestre* (L.) Sch. et Thell as a synonym for *Anthriscus sylvestris* (L.) Hoffm. [12,13,14]. However, Spalik showed in a phyllogeny study regarding *Anthriscus* genus classification, that *A. sylvestris* has at least five subspecies: subsp. *sylvestris*, subsp. *nemorosa* (M.-Bieb) Kozo-Pol., subsp. *fumarioides* Spalik, subsp. *alpina* (Villar) Gremli and subsp. *torquata* Koch (Reduron & Spalik) [15]. Before the revision of the *Anthriscus* classification performed by Spalik, subsp. *nemorosa* and *fumarioides* were considered distinctive species (*A. nemorosa* Bieb. Sprengel and *A. fumarioides* (Waldst. & Kit.) Sprengel) [16]. The two species are still often confused in scientific literature [17]. A revised list published by Magnusson comprises the following synonyms: *Chaerefolium silvestre* (L.) Sch. et Thell., *Chaerophyllum sylvestre* L., *C. nemorosum* Bieb., *Chaerefolium sylvestre* (L.) Thell., *Cerefolium sylvestre* Besser., *Anthriscus nemorosa* (Bieb.) Spreng [18]. All subspecies have 2n = 16 chromosomes [9,15,18]. The common names includes cow parsley, keck, wild beaked parsley, wild chervil, wild parsley and woodland chervil [18].

### 2.2. Etymology

The etymology of the genus name “*Anthriscus*” is of uncertain origin. The term originates from Greek, from chervil or southern chervil (*Anthriscus cerefolium* (L.) Hoffm.) [19], a plant known since antiquity as a spice and probably was used in the laudanum formula along with *Cistus* species [20,21]. Therefore, *Anthriscus sylvestris* means “chervil from the woods” [22].

### 2.3. Distribution and Habitat

*A. sylvestris* L. Hoffm. (Apiaceae) is a common wild plant in northwest Europe, in parts of North America, Africa, Asia and New Zealand. Its native range includes Europe and temperate Asia [18]. In Europe it is common in most countries, although it is rare in the Mediterranean region [2] and in Iceland, Faroe Islands and Greenland, where is regarded as an alien species [23,24]. The species was also introduced in North America, Canada, Alaska, central and southern Africa and New Zealand [25,26]. 

It is characteristically found in hedgerows and road verges but also on woodland edges and in neglected pastures and hay meadows. Depending of the edaphic conditions or population characteristics, the plant has been reported as an annual, biennial or short-lived perennial [27,28,29]. However, the species is always monocarpic, regardless of the period of growth and development [30,31]. Thus, annual subspecies and varieties are found in drier habitats and occupy lower montane regions [15]. Moreover, the species can adapt to several different types of habitat, such as Subtropical zones [32], adaptation which however will prologue the life cycle and, thus alter the growing and development of different organs [33]. The species prefers moist to mesic soils with a pH value ranging from 6.2 to 7.0 [34]. Not being pretentious to light conditions, it can grow under light conditions ranging from semi-shade to fully open sites [35,36].

### 2.4. Botanical Description

*A. sylvestris* is parented by buds of the basal leaves which develop roots after fruit ripening [37]. The root is thick and can reach up to 2 m [38]. The plant has erect stems of 0.3 to 1.5 m tall with short, eglandular hairs. Leaves are green to pale reddish, triangular in outline, 3-pinnate, and acute at the apex. Flowers are grouped together in glabrous inflorescences of compound umbels, with (3–) 6–12 rays, with 4–6 bracteoles and without bracts [37]. The calyx, initiated at the beginning of the development, has a strong tendency for reduction, therefore, the sepals are reduced [39]. The flower is composed of five unequal, creamy white petals, five stamens with white filaments and yellow or cream anthers, and gynoecium with two carpels and two divergent styles [37,40]. The fruits, green at the beginning, turn brown to reddish when they ripen. The mericarps are glabrous, smooth, with a short beak, and present five slight ribs [41].

## 3. Ethnomedicinal Uses

Wild chervil was used among other wild species of the Apiaceae family as “parsley” in Northern Europe. The aerial parts were used as a cure for kidney stones, along with parsely (*Petroselinum crispum* (Mill.) Fuss). Although the traditional use of *A. sylvestris* in Europe was rather the result of its confusion with other Apiaceae species than ethnobotanical knowledge [42], Deforce has shown that the plant was common in the antiquity [21]. The aerial parts were also used in Ireland and Tunisia to treat headaches [42,43] and in Serbia as a diuretic and tonic [44]. In Asia, the roots have traditionally been used as an antipyretic, an analgesic, a diuretic, and a cough remedy [3,45]. In India, the plant is still used by the indigenous communities to treat rheumatism and other inflammatory ailments [46]. In Anatolia, the fruits were used as spice for cheese along other aromatic herbs [47].

## 4. Phytochemical Profile

Chemical composition of *A. sylvestris* was revealed in several phytochemical studies performed on fresh and dried leaves, flowers, fruits and roots. The major classes of phytochemicals include terpenoid compounds, phenolic compounds and flavonoid lignans [48,49]. Among them, the lignans were the most studied because their implications in cytotoxic activity and their similarity with other well documented compounds isolated from *Podophyllum* species. Except for these classes of phytochemicals, in *A. sylvestris* were also found compounds belonging to carotenoids, sterols (β-sitosterol), anthocyanins and vitamins [44,49,50].

### 4.1. Root

The volatile components of fresh roots were isolated through hydrodistillation and analysed by GC and GC-MS. The chemical profile of the root and leaf oils correspond, with only few differences. The monoterpene fraction represents the major constituent (69%) and β-phellandrene (45.5%), *Z*-β-ocimene (16.9%) and α-pinene (4.6%) are its major components [51].

Several coumarins were identified in the methanol soluble fraction of the roots of *Anthriscus sylvestris*: scopoletin, isoscopoletin and bergapten (5-methoxypsoralen) [52]. In the chloroform fraction of the methanolic extract from the dried roots two phenylpropene derivatives were identified: 1-(3′-methoxy-4′,5′-methylenedioxyphenyl-1-methoxy-2-propene and elemicin, one polyacetylene derivative: falcarindiol, and several lignans: deoxypodophyllotoxin (anthricin), (−)-deoxypodorhizone, anthriscusin and nemerosin [4]. The occurrence of another polyacetylene derivative, falcarindiol-3-acetate, has been reported by Kramer *et al.* [53].

Using HPLC combined with electrospray tandem mass spectrometry and NMR spectroscopy techniques, Hendrawati *et al.* found nine lignans and five related structures in the roots of *A. sylvestris*. The major compounds are deoxypodophyllotoxin, podophyllotoxone, yatein, anhydropodorhizol, 1-(3′-methoxy-4′,5′-methylenedioxyphenyl-1-methoxy-2-propene and (2*E*)-3-(7-methoxy-1,3-benzodioxol-5-yl)-2-propen-1-yl 2-methyl-4-[[(2*Z*)-2-methyl-1-oxo-2-buten-1-yl]oxy]-2-butenoate. α-Peltatin, β-peltatin, isopicropodophyllone, β-peltatin methylether, (*Z*)-2-angeloyloxymethyl-2-butenoic acid, anthriscinol methylether, and anthriscrusin are present in lower concentrations [54]. The low concentration of α- and β-peltatin might be due to the fact that the roots were collected after the plant flowed and might be due to metabolic turnover [55]. Podophyllotoxin have been reported in *A. sylvestris* in trace amounts, under 0.01 μg/mg of dry product. The study identified also small quantities of arctigenin, dimethylmatairesinol, dimethylthujaplicatin, 7-hydroxyyatein and 7-hydroxy-anhydropodorhizol [56]. Jeong *et al.* identified sylvestrin in the hexane-soluble fraction of the methanolic extract. Picropodophyllotoxin was identified in the chloroform fraction [52].

### 4.2. Aerial Parts

The volatile components of fresh leaves, obtained through hydrodistillation, were analysed by GC and GC-MS. The monoterpene fraction represents the major constituent (70%). β-phellandrene (38.8%), β-myrcene (16.7%), sabinene (6.2%) and *Z*-β-ocimene (5.4%) were identificated as main components [51]. The phytochemical components of *Anthriscus sylvestris* flowers and leaves include also benzyl alcohol, 2-phenyl-ethanol, eugenol, saligenol, (*Z*)-3-hexenol, α-pinene-7-ol acetate and β-farnesene [57]. In another study, Nickavar *et al.* have identified 41 compounds from aerial parts, mainly monoterpenoids [17]. The authors have found (*E*)-nerolidol to be the major compound (41.7%) [17]. Other compounds found were: β-elemene (13.0%), α-zingiberene (9.9%), germacrene-D (5.0%), (*E*,*E*)-α-farnesene (3.9%), α-pinene (3.7%) and (*E*)-caryophyllene (2.3%). The authors explained the variation regarding the composition of the volatile oil by the variability of the plant species and the existence of different chemotypes [17].

Dried aerial parts of *A. sylvestris* were extracted for 48 h with ethanol (70%) at 23 °C. The ethanolic extract demonstrated antioxidant activity. Separation and identification of antioxidant components by thin-layer and column chromatography and spectral analysis demonstrated that quercetin and apigenin appeared to be the main flavonoid species and rutin was one of the major quercetin glycosides [58]. Dall’Acqua *et al.* analyzed the crude methanol extract of aerial parts and found that the antioxidant active fractions contained mainly luteolin-7-*O*-glucoside and chlorogenic acid [59]. Luteolin-7-*O*-glucoside (cynaroside) was confirmed as the dominant polyphenolic species by the work of Žemlička *et al.* Partially wilted flowers and stems of *A. sylvestris* were macerated twice with acetone for 24 h. The solvent was removed under vacuum, and the low-boiling fractions were separated by hydrodistillation. The residue was extracted with ethyl acetate to remove all lipid fractions and purified to yield 0.124 g cynaroside/kg of fresh plant [60]. Cynaroside was also isolated from the flowers of *Anthriscus sylvestris* [61]. In another study, Abdulmanea *et al*. found in leaves by HPLC-MS besides quercetin, rutin and apigenin, the flavone quercetin-3-*O*-glucoside (isoquercetin) and several isoflavones: daidzin, daidzein, genistin (genistein 7-glucoside), sissotrin (biochanin A 7-*O*-β-d-glucoside) and formononetin [62].

The content lignans in the aerial parts of *A. sylvestris* is significantly lower than in the roots. The lignan profile of the aerial parts of *A. sylvestris* was analyzed using hot methanol and treated β-glucosidase. The lignans deoxypodophyllotoxin, yatein, secoisolariciresinol, lariciresinol, matairesinol, hinokinin, pluviatolide and nemerosin were identified [63].

**Figure 1:**
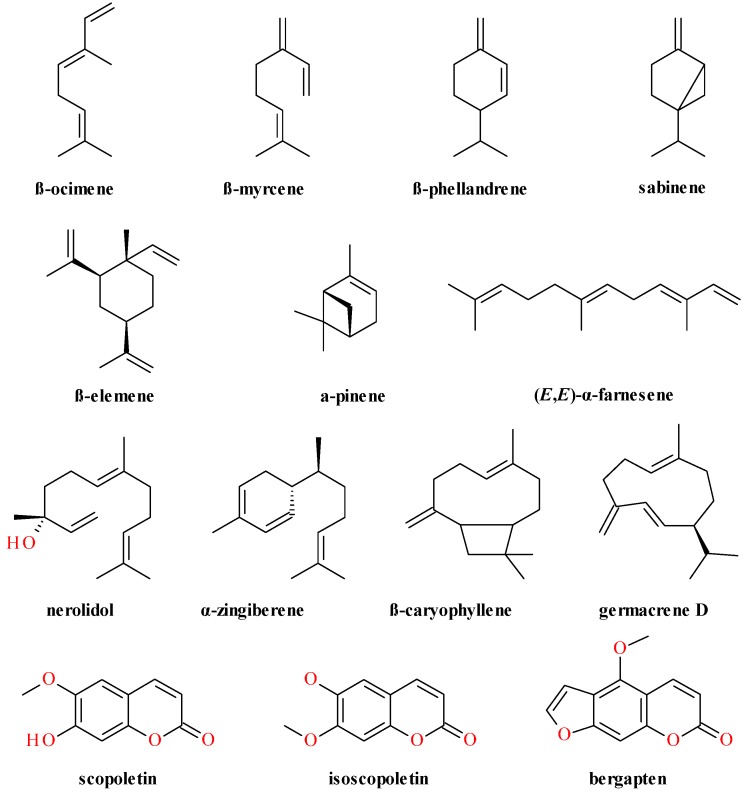
Volatile compounds from *Anthriscus sylvestris* (L.) Hoffm.

The presence of fitosterols was analyzed. Plants contained up to 0.03% α-sitosterol in their aerial parts and clear phenotypic differences in the α-sitosterol contents were reported [56]. In Figure 1 the chemical structures for the main volatile compounds found in roots and aerial parts of *A. sylvestris* are presented.

**Figure 2:**
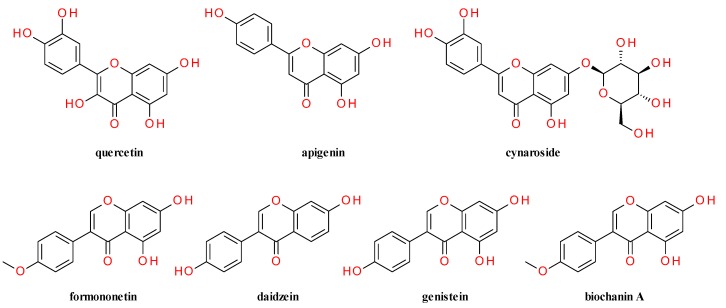
Flavonoids and isoflavones identified in *Anthriscus sylvestris* (L.) Hoffm.

The structure of the main the flavonoids and isoflavones found in aerial parts of *A. sylvestris* are presented in Figure 2.

### 4.3. Fruits

The constituents in the fruit of *Anthriscus sylvestris* were investigated and four lignans: deoxypodophyllotoxin, yatein, (−)-morelensin, and (−)-hinokinin were isolated. Morelensin and hinokinin were reported only in the fruits, and not in the root of the plant. Other constituents were identified as morinin L, falcarindiol, 1′-hydroxymethyleugenol, 3′,4′-dimethoxycinnamyl (*Z*)-2-angeloyloxymethyl-2-butenoate and 3',4'-dimethoxycinnamyl (*Z*)-2-tigloyloxymethyl-2-butenoate [64].

### 4.4. Lignans Profile and Content

The lignan profile and content is highly influenced by environmental factors. It has been reported that the content of deoxypodophyllotoxin is at least 2-fold higher at high than low altitudes both in aerial and in root parts of *A. sylvestris*. In the aerial part the content of deoxypodophyllotoxin was measured as 0.13% at 900 m and 0.33% at 1200 m. The root of the plant harvested from an altitude of 900 m contained 0.38% deoxypodophyllotoxin, compared with 0.78% in those from 1200 m [59].

The content of deoxypodophyllotoxin in the aerial parts of *A. sylvestris* collected from the wild was small, and the total lignan content was significantly lower than in the roots. In the aerial parts of the indoor grown plants the concentration of the lignans in aerial parts were significantly higher than the concentrations found in the roots. The total lignan content of the roots of plants cultivated indoors was comparable to the concentrations found in the wild [64]. The root mass of the outdoor plants was up to eight times, and the aerial mass was up to 30 times higher than that of the indoor plants. The deoxypodophyllotoxin yield of the outdoor plants was up to eight times higher in the aerial part and up to five times higher in the root part, compared with the indoor plants [65].

For field grown plants the highest deoxypodophyllotoxin content was found in March (second year): 0.15% (dry weight) in roots and 0.03% in aerial parts. For plants grown in a climate room, the highest concentration (0.14%) was observed in April (second year) in the roots and in July (first year) in the aerial parts (0.05%). For the optimal isolation yield of deoxypodophyllotoxin the roots are the most suitable part and the best harvest time is March (second year) for outdoor plants and April (second year) for indoor plants [66].

A various number of plant species, mainly belonging to the Cupressaceae, Berberidaceae, Euphorbiaceae, Menispermaceae and Hernandiaceae families, contain deoxypodophyllotoxin and have an important role in traditional medicine. In Table 1 are presented the plants with a known content of deoxypodophyllotoxin. Deoxypodophyllotoxin was identified in other plant species in trace or small amounts, among which, we mention *Thuja occidentalis* (Cupressaceae) [67], *Juniperus* species (Cupressaceae): *J. rigida* [68], *J. scopulorum* [69], *J. procumbens* [70], *Illigera luzonensis* (Hernandiaceae) [71], *Hernandia* species (Hernandiaceae): *H*. *nymphaeifolia* [72], *H*. *peltata* [73], *H*. *sonora* [74], *Bursera fagaroides* (Burseraceae) [75] and *Pulsatilla koreana* (Ranunculaceae) [76].

**Table 1:** Plants containing deoxypodophyllotoxin.

Genus and Family	Species	Part Used and Content
*Juniperus* (Cupressaceae)	*J. taxifolia*	Leaves (0.004%) [77]
	*J. sabina*	Leaves (0.125%) [78,79]
	*J. davurica*	Leaves (0.727%) [69]
	*J. communis*	Leaves and stems (0.007%) [80,81] Stems (0.008–0.017%) [70]
	*J. blaaws*	Stems (0.008%) [70]
	*J. x-media*	Stems (0.024%) [70]
	*J. squamata*	Stems (0.025%) [70]
	*J. recurva*	Stems (0.011%) [70]
	*J. bermudiana*	Leaves (0.44%) [82]
*Callitris* (Cupressaceae)	*C. endlicheri*	Leaves (0.392%–0.577%); stems (0.198%); cones (0.053%) [83].
	*C. columellaris*	Leaves (0.062%) [84,85]
	*C. rhomboidea*.	Leaves (0.178%–0.350%); stems (0.119%); cones (0.066%) [83]
	*C. preissii*	Leaves (0.010%–0.015%); stems (0.008%); cones (0.003%) [83]
	*C. drummondii*	Leaves (0.010%–0.015%) [83,86]; stems (0.008%); cones (0.003%) [83]
*Bridelia* (Euphorbiaceae)	*B. ferruginea*	Roots (0.001%) [87]
	*B. microphylla*	Stems (0.09%) [88]; fruit [89]
	*B. morelensis*	Stems (0.31%) [84,88]
	*B. permollis*	Stems (0.004%) [84,88]
*Macrococculus* (Menispermaceae)	*M. pomiferus*	Dried stems (0.001%) [90]
*Podophyllum* (Berberidaceae)	*P. peltatum*	Roots (0.023%) [84,88,91]
*Sinopodophyllum* (Berberidaceae)	*S. hexandrum*	Roots and rhizomes (0.008%) [92,93]
*Dysosma* (Berberidaceae)	*Dysosma versipellis*	Roots (0.504%) [94]

**Figure 3:**
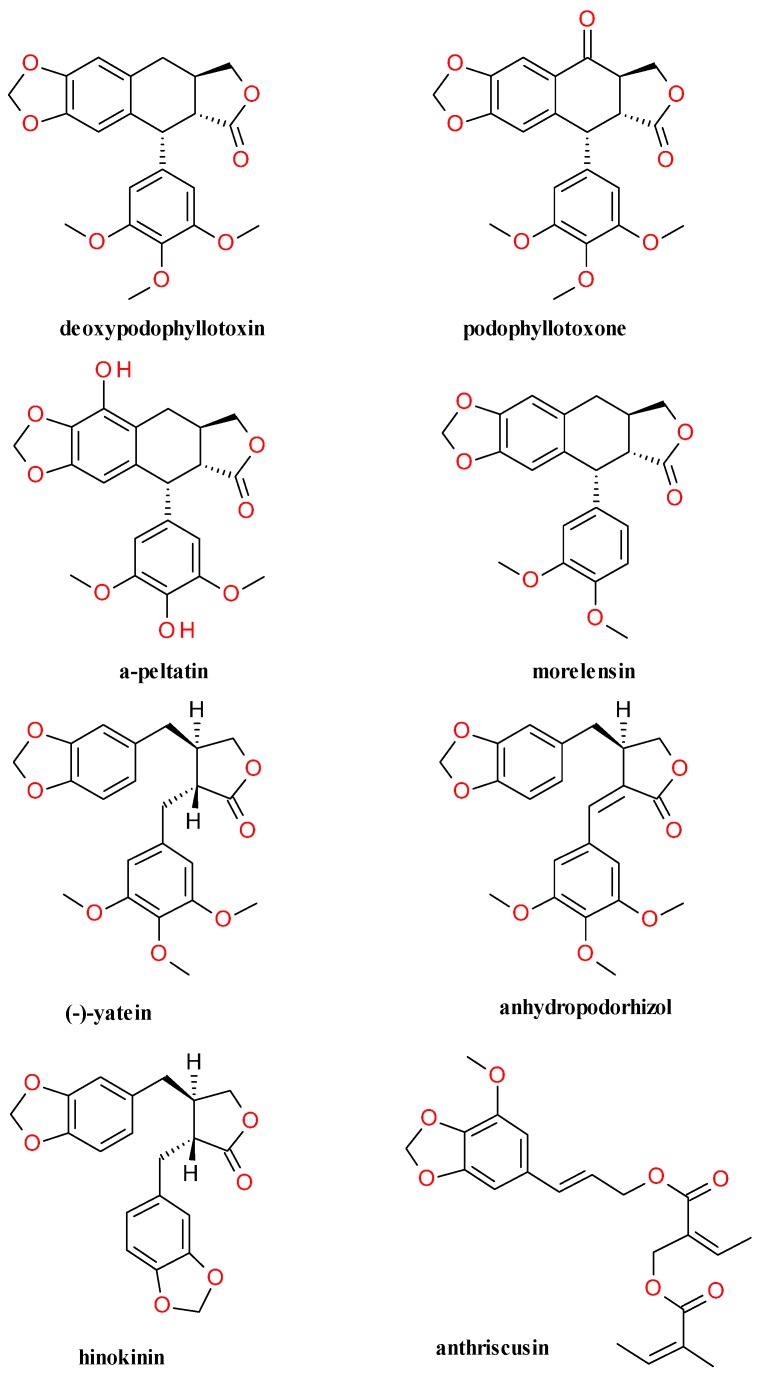
The major lignans found in *Anthriscus sylvestris* (L.) Hoffm.

The analysis of the other major lignans’ content in the root compared to the deoxypodophyllotoxin concentration revealed relative level of 26 ± 17% for yatein and of 29 ± 16% for anhydropodorhizol [64]. The chemical structures of the major lignans found in *A. sylvestris* are presented in Figure 3.

The biosynthesis pathway of the lignans in *A. sylvestris* starts from coniferyl alcohol leading to pinoresinol which is transformed to lariciresinol and then to secoisolariciresinol, and subsequently converted to matairesinol [63]. Sakakibara *et al.* proposed the transformation of matairesinol to thujaplicatin followed by the methylation of thujaplicatin to 4,5-*O*,*O*-dimethylthujaplicatin leading to yatein and afterwards to deoxypodophyllotoxin in *A. sylvestris* [95]. Ragamustari *et al*. demonstrated the methylation of thujaplicatin by plant’s *O*-methyltransferases [96]. An alternative pathway is the conversion of matairesinol to deoxypodophyllotoxin via anhydropodorhizol [54].

## 5. Pharmacology

### 5.1. Antitumor Activity

Ikeda *et al.* performed one of the first studies to evaluate the anti-proliferative effects of the root and aerial part of *A. sylvestris* and showed a high *in vitro* inhibitory activity against MK-1, HeLa, and B16F10 cell growth. The activity was found only in the chloroform soluble fractions [4]. Activity-guided fractionation of the roots resulted in the isolation and characterization of five cytotoxic compounds: deoxypodophyllotoxin, falcarindiol, angeloyl podophyllotoxin, morelensin, and bursehernin [97].

#### 5.1.1. Deoxypodophyllotoxin

Podophyllotoxin disrupts the function and dynamics of microtubules by binding to the tubulin dimer [98] and at higher concentrations inhibits the nucleoside transport through the cell membrane [99]. Podophyllotoxin binds to tubulin and prevents the formation of the mitotic spindle, resulting in an arrest of the cell division process in metaphase [100]. It is effective in the treatment of Wilms tumours, various genital tumours and in non-Hodgkin lymphomas [101], but the clinical trials were unsuccessful due to severe gastrointestinal side effects [102].

The identification of deoxypodophyllotoxin as the main lignan in the root and ground parts of the plant was correlated with the findings of previous studies in which the antitumor effects of deoxypodophyllotoxin were evaluated as podophyllotoxin’s congener [103,104]. Deoxypodophyllotoxin binds directly to tubulin, resulting in the inhibition of microtubule assembly by inhibiting tubulin polymerization and induces G2/M arrest and accumulation of cells in sub-G1 phase followed by apoptosis [45].

Treatment of SGC-7901 cells with deoxypodophyllotoxin concentrations in the range of 25–100 nM resulted in a strong tumor inhibition by time- and dose-dependent decrease in Cdc2 and Cdc25C expression levels and the augmentation of cyclin B1 level [105]. Deoxypodophyllotoxin induces apoptosis in HeLa cells through multiple cellular processes, involving the activation of ataxia-telangiectasia mutated kinase, upregulation of p53 and Bax, activation of caspase-3 and -7, and accumulation of the phosphatase and tensin homolog (PTEN) resulting in the inhibition of the Akt pathway [106]. The inhibition of Akt by deoxypodophyllotoxin is demonstrated by the inhibition of mTOR kinase activity in a time- and dose-dependent manner [107].

Wu *et al.* studied the effect of deoxypodophyllotoxin on lung carcinoma cells NCI-H460 and demonstrated that it significantly inhibits the cell proliferation with IC_50_ of 11.4 nM after a 24 h exposure. Deoxypodophyllotoxin triggered necroptosis, autophagy, loss of plasma membrane integrity, the elevation of reactive oxygen species levels, and a specific inhibition of necroptosis via necrostatin-1. In a nude mice xenograft model, administration of 20 mg/kg deoxypodophyllotoxin inhibited the tumor growth by 69.6% [108].

A functioning vascular supply is essential for solid tumor growth and metastases, which means that targeting tumor vasculature can be an ideal solution for antitumor drug discovery. The anti-angiogenic and vascular disrupting activities of deoxypodophyllotoxin were examined in the rat aortic ring test and chick chorioallantoic membrane assay. Deoxypodophyllotoxin induced cytoskeleton reorganization in endothelial cells, which likely contributed to the anti-angiogenic effect at non-cytotoxic concentrations. Treatment with 40 nM of deoxypodophyllotoxin disrupted capillary-like networks and newly formed vessels from rat aortic rings demonstrating potent anti-angiogenic and vascular disrupting effects [109].

#### 5.1.2. Semisynthetic Derivatives

**Figure 4:**
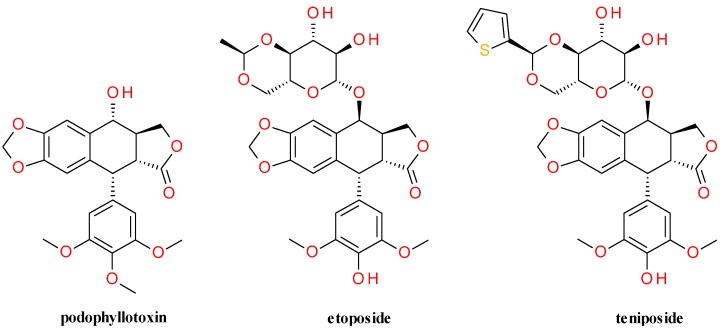
The structures of podophyllotoxin, etoposide and teniposide.

Deoxypodophyllotoxin and podophyllotoxin contain both a five-ring system, of which the methylenedioxy and 3,4,5-trimethoxyphenyl rings are reported to be essential for its anticancer activity [101]. Extensive structural modifications, particularly at the C-4 and C-4′ positions have led to the development of many semisynthetic derivatives. Among them, etoposide, teniposide, etopophos, GL331 and TOP-53 are currently used in the clinic for the treatment of a variety of malignancies including, lung and testicular cancers, lymphoma, and glioblymphocytic leukemia [110]. These derivatives display anticancer effects through a mechanism of action entirely different from that of their parent compounds, binding to DNA topoisomerase II during the late S and early G2 cell cycle stages [111]. Figure 4 presents the structures of etoposide and teniposide, the most used semisynthetic epipodophyllotoxin derivatives.

### 5.2. Antimicrobial Activity

The whole plant was extracted with methanol under reflux and fractioned with *n*-hexane, methylene chloride, ethyl acetate, and *n*-butanol. The antibacterial activities of the fractions were tested against *Escherichia coli*, *Staphylococcus aureus*, and *Helicobacter pylori* using the disc agar diffusion method. The *n*-hexane and methylene chloride fractions showed a stronger antibacterial activity against *S. aureus* than other fractions [112].

Deoxypodophyllotoxin was tested and revealed a significant antimicrobial effect against both Gram-positive and Gram-negative bacteria, except on *Escherichia coli*. It has a more pronounced activity on Gram-positive than on Gram-negative bacteria, but is less potent than podophyllotoxin in this respect [70]. Deoxypodophyllotoxin proved to be a highly potent and selective inhibitor of herpes simplex virus types 1 and 2 in MRC-5 cells. In contrast, it was found to have no antiviral effect against influenza A virus, respiratory syncytial virus or human cytomegalovirus in doses that are not toxic to the cells [6].

### 5.3. Anti-Inflammatory Activity

The dried roots of *A. sylvestris* have been used in traditional medicine as an antipyretic, analgesic and cough remedy. Deoxypodophyllotoxin, the main lignan present in the root, caused significant inhibition of paw edema development in the carrageenan-induced inflammation test (66.3% ± 4.4%) similar with that produced by indomethacin (61.5% ± 2.5%), a standard anti-inflammatory drug [7]. 

In the attempt to understand the mechanisms accounting for its anti-inflammatory effects, deoxypodophyllotoxin was isolated from the methanol-dichloromethane extract of *A. sylvestris* root. Deoxypodophyllotoxin inhibits cyclooxygenase (COX) 1 and 2 prostaglandin D2 (PGD2) generation, in a concentration-dependent manner with IC_50_ values of 1.89 mM and 65.3 mM, respectively. It inhibits also COX-1 and 2 dependent conversion of the exogenous arachidonic acid to PGD2 in a dose-dependent manner with an IC_50_ values of 0.01 mM and 12.1 mM and it inhibits in a dose dependent manner the production of leukotriene C4. The antipyretic and analgesic activity of *A*. *sylvestris* extracts could be attributed at least in part to the dual inhibition of COX-2 and 5-lipoxygenase [113].

The anti-inflammatory effect of deoxypodophyllotoxin is also explained by its capacity to suppress nitric oxide (NO) generation through the inhibition of NF-κB activation, a critical inflammatory transcription factor [114].

Deoxypodophyllotoxin was tested in a passive cutaneous anaphylaxis (PCA) assay by administering deoxypodophyllotoxin intraperitoneally (1.0 to 10 mg/kg) and intravenously (0.25 to 1.0 mg/kg) to laboratory rats. Deoxypodophyllotoxin inhibited in a dose-dependently manner the PCA. The PCA inhibitory activity of deoxypodophyllotoxin was stronger than those of prednisolone and indomethacin, suggesting that deoxypodophyllotoxin may be beneficial in regulating the immediate-type allergic reactions [115].

### 5.4. Antioxidant Activity

An antioxidant-guided fractionation of the crude methanol extract from the aerial parts of *A. sylvestris* was performed using the DPPH test. The active fractions contained mainly luteolin-7-*O*-glucoside and chlorogenic acid. The antioxidant properties of both crude extract and isolated compounds were also investigated with the Briggs-Rauscher reaction [59]. Separation of the components of the ethanol/water (7/3) extracts by thin-layer and column chromatography demonstrated that quercetin, apigenin and rutin are the main antioxidant species [58].

## 6. Biotechnology Applications

The chemical synthesis of podophyllotoxin is considered to be complicated and the availability of the compound from plants has its limitations. Deoxypodophyllotoxin is structurally closely related to podophyllotoxin, and can be converted into epipodophyllotoxin, the starting material for the synthesis of the anticancer drugs etoposide and teniposide [116].

### 6.1. Callus Tissues

Plant tissue culture, or the aseptic culture of cells, tissues, organs, and their components under defined physical and chemical conditions *in vitro*, is an important tool in both basic and applied studies as well as in commercial application [117]. Calli of *A. sylvestris* did not produce deoxypodophyllotoxin and whole plants were required in order to achieve the differentiated forms to produce deoxypodophyllotoxin for metabolic engineering purposes [118]. In the cell suspension cultures only trace amounts of deoxypodophyllotoxin were detected. In the feeding experiments with suspended cells, deoxypodophyllotoxin was converted into podophyllotoxin, yielding significantly higher concentration than measured in whole plants [56].

### 6.2. Heterologous Expression System

A new alternative biotechnological method is the conversion of deoxypodophyllotoxin isolated from *A. sylvestris* roots into epipodophyllotoxin, the diastereoisomer of podophyllotoxin. This conversion is performed in yields up to 90% by *Escherichia coli* DH5α transformed with recombinant human liver cytochrome P450 3A4. There was no detectable production of epipodophyllotoxin or podophyllotoxin by CYP1A2 and CYP2C9 enzymes [119]. Later studies have shown a mechanism-based inhibition of CYP3A4 enzyme by deoxypodophyllotoxin and epipodophyllotoxin with an important influence in the application of the described bioconversion system [120].

Deoxypodophyllotoxin was also converted to epipodophyllotoxin in the high yield using *Penicillium* F-0543. Eight other type species of *Penicillium* converted deoxypodophyllotoxin to epipodophyllotoxin with various success. Some species of *Aspergillus niger* were tested, but the yields were very low [121].

## 7. Conclusions

*Anthriscus sylvestris* Hoffm. is a common and fast-growing plant rich in active compounds potentially useful in treatment of cancer and of inflammatory diseases. Its main active compound, deoxypodophyllotoxin, has antiproliferative, antitumor, antiviral, anti-inflammatory, and anti-allergic properties. Deoxypodophyllotoxin also might be used as a precursor to synthesize epipodophyllotoxin, the starting material for anticancer drugs such as etoposide and teniposide. Its ability to grow rapidly and the high adaptability to grow in almost any type of soil makes *Anthriscus sylvestris* a highly valuable source of both lignan derivatives and flavonoid compounds for use in the pharmaceutical industry. The use of biotechnology tools can create new opportunities to produce metabolically engineered *A. sylvestris* and valuable new drug compounds.
